# Induction of PNAd and N-acetylglucosamine 6-O-sulfotransferases 1 and 2 in mouse collagen-induced arthritis

**DOI:** 10.1186/1471-2172-7-12

**Published:** 2006-06-13

**Authors:** Jiwei Yang, Steven D Rosen, Philip Bendele, Stefan Hemmerich

**Affiliations:** 1 Thios Pharmaceuticals Inc., P.O. Box 20010, Oakland, CA 94620, USA; 2 Department of Anatomy and Program in Immunology, University of California, Box 0452, San Francisco, CA 94143, USA; 3 Bolder BioPATH Inc., University of Colorado, CB 345, Boulder, CO 80309, USA; 4 Current Address: Geron Corporation, 230 Constitution Drive, Menlo Park, CA 94025, USA; 5 Current Address: Y's Therapeutics Inc., 866 Malcolm Rd., Suite no.100, Burlingame, CA 94010, USA

## Abstract

**Background:**

Leukocyte recruitment across blood vessels is fundamental to immune surveillance and inflammation. Lymphocyte homing to peripheral lymph nodes is mediated by the adhesion molecule, L-selectin, which binds to sulfated carbohydrate ligands on high endothelial venules (HEV). These glycoprotein ligands are collectively known as peripheral node addressin (PNAd), as defined by the function-blocking monoclonal antibody known as MECA-79. The sulfation of these ligands depends on the action of two HEV-expressed N-acetylglucosamine 6-O-sulfotransferases: GlcNAc6ST-2 and to a lesser degree GlcNAc6ST-1. Induction of PNAd has also been shown to occur in a number of human inflammatory diseases including rheumatoid arthritis (RA).

**Results:**

In order to identify an animal model suitable for investigating the role of PNAd in chronic inflammation, we examined the expression of PNAd as well as GlcNAc6ST-1 and -2 in collagen-induced arthritis in mice. Here we show that PNAd is expressed in the vasculature of arthritic synovium in mice immunized with collagen but not in the normal synovium of control animals. This de novo expression of PNAd correlates strongly with induction of transcripts for both GlcNAc6ST-1 and GlcNAc6ST-2, as well as the expression of GlcNAc6ST-2 protein.

**Conclusion:**

Our results demonstrate that PNAd and the sulfotransferases GlcNAc6ST-1 and 2 are induced in mouse collagen-induced arthritis and suggest that PNAd antagonists or inhibitors of the enzymes may have therapeutic benefit in this widely-used mouse model of RA.

## Background

Chronic inflammatory diseases such as rheumatoid arthritis (RA), asthma, inflammatory bowel disease (IBD), and multiple sclerosis still pose a large unmet medical need despite recent therapeutic advances such as inhaled steroids (asthma) or TNFα antagonists (RA and IBD). Thus, significant subpopulations of patients, in particular those with severe disease, respond only poorly to these treatments [1,2]. Furthermore, patients treated with TNFα antagonists are at risk for serious infections [3]. Therefore, modulation of leukocyte-endothelial adhesion, an obligatory step in the recruitment of inflammatory cells to lesions, has been widely considered as an alternative and perhaps complementary approach for therapy of chronic inflammation [4,5]. One of the molecules involved in leukocyte trafficking is L-selectin, a member of the selectin family of cell adhesion molecules, which is expressed on leukocytes [6]. During the process of lymphocyte homing to lymph nodes, L-selectin mediates rolling of lymphocytes on high endothelial venules (HEV). This is the first step in a cascade of adhesion and signaling events that culminate in the recruitment of both naïve and central memory lymphocytes into lymph nodes [7]. The major class of ligands recognized by L-selectin consists of a family of sialomucins defined by the adhesion-blocking antibody known as MECA-79. Collectively these ligands are termed peripheral node vascular addressin (PNAd) [8] or sulfoadhesin [9]. One of the shared features of these ligands is 6-O-sulfated N-acetylglucosamine, which is essential for antibody as well as L-selectin binding [10-12]. This modification is found on 6-sulfo sLe^x ^which is a minimal recognition determinant for L-selectin [13,14]. The 6-O-sulfation of N-acetylglucosamine of PNAd components occurs in the Golgi compartment and is catalyzed by Golgi-associated N-acetylglucosamine 6-O-sulfotransferases (GlcNAc6STs) [15-17]. Using gene deletion by homologous recombination in mice, we have shown that GlcNAc6ST-2, the high endothelial cell restricted N-acetylglucosamine 6-O-sulfotransferase also known as HEC-GlcNAc6ST, LSST, or GST-3 (gene name *chst4 *in mouse) is largely responsible for the GlcNAc-6-sulfation of PNAd and contributes substantially to L-selectin ligand activity and MECA-79 reactivity [18,19]. A related enzyme known as GlcNAc6ST-1 or GST-2 [20] (gene name *chst2*) also contributes to sulfoadhesin biosynthesis but to a lesser degree [17,21,22].

While being constitutively expressed in the HEV of lymph nodes and other secondary lymphoid organs, the induction of PNAd has been reported in activated vessels in synovial biopsies from RA patients [23-26], in a model of Lyme disease arthritis in severe combined immunodeficient (SCID) mice infected with *Borrelia burgdorferi (B. burgdorferi) *[27], as well as in many other inflammatory lesions [28]. In addition, extralymphoid induction of PNAd in inflammatory lesions was shown to correlate with the de novo expression of GlcNAcST-2 in human RA [25] as well as animal models of autoimmunity [16,29]. These findings suggested, that blockade of PNAd, either directly, or indirectly through inhibition of the responsible sulfotransferase(s), might be efficacious for anti-inflammatory therapy [30]. As any drug discovery effort relies on robust and predictive animal models, we have studied the expression of sulfoadhesin and GlcNAc6ST-1 and -2 in murine collagen-induced arthritis (CIA), a widely used animal model which is predictive for therapeutic benefit in human rheumatoid arthritis [31-33]. Our data show that PNAd is expressed in this model in arthritic but not in healthy synovial tissue, and that GlcNAc6ST-1 and 2 are induced in arthritic synovium at the transcript level for both enzymes and at the protein level for at least GlcNAc6ST-2.

## Results

### GlcNAc6ST-1 and -2 transcript are upregulated in arthritic but not in normal synovium

**Figure 1:**
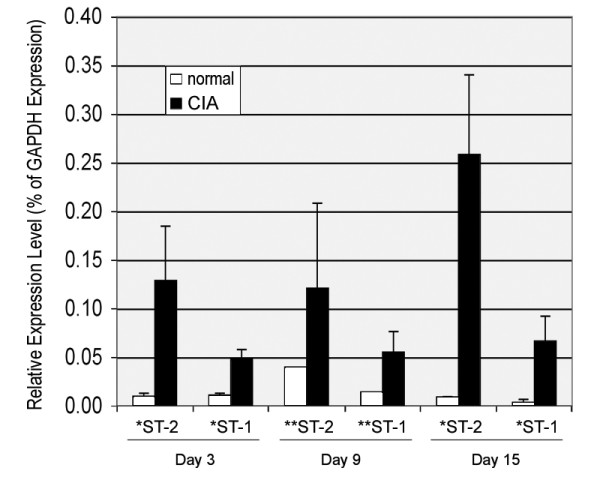
**Expression levels of GlcNAc6ST-1 (ST-1) and GlcNAc6ST-2 (ST-2) in arthritic versus control joints in mouse CIA**. * p < 0.05, ** only one data point for normal. Error bars represent standard deviations.

In order to investigate the potential relevance of GlcNAc6ST-1 and -2 in the CIA model, we compared the expression of transcripts for these enzymes in arthritic and control synovial tissue by quantitative PCR. We also measured transcripts levels for genes whose expression is known to be upregulated in this model (IL-1β and IL-10) as well as transcript levels for GAPDH, a house keeping gene whose expression does not change in diseased joints [34]. All expression data were normalized to GAPDH levels (% of GAPDH expression). As shown in Fig. 1, GlcNAc6ST-2 transcripts and to a lesser degree GlcNAc6ST-1 transcripts were upregulated in diseased joints compared to control joints at all three time points examined. The mean fold-induction of GlcNAc6ST-2 and GlcNAc6ST-1 in diseased joints as compared to normal joints (all time points pooled) was 11-fold and 6-fold, respectively (p < 0.0005). Although levels of GlcNAc6ST-2 transcripts in arthritic joints trended to be higher at day 15 than on day 3 or 9, this difference was not statistically significant. Levels of inflammatory cytokines IL-1β and IL10 were also elevated in diseased as compared to control joints (80-fold and 7-fold, respectively) in agreement with previous reports [35].

### PNAd is induced in arthritic but not in control synovium

**Figure 2:**
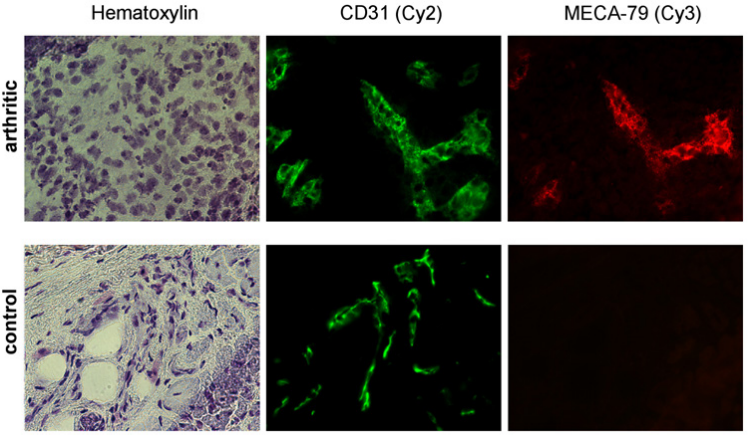
**Induction of PNAd in arthritic synovium at day 3**. Sections of knee or ankle joint synovial tissue (obtained at days 3, 9, and 15) and control tissue (from same times and joints) were stained with a CD31 antibody (Cy-2, green), MECA-79 (Cy3, red) and with hematoxylin for bright field visualization. The depicted two-color fluorescence micrographs (400-fold magnification) were taken on sections from day 3 and are representative for the other sections from this time. Sections from days 9 and 15 of the model stained similarly; however, the staining intensity with MECA-79 and the fraction of vessels staining with MECA-79 appeared to be somewhat less than in sections from day 3 (quantitative image and statistical analysis was not performed).

We also probed for presence of PNAd in arthritic and normal synovium by performing immunofluorescence (IF) on cryostat-cut sections of synovial tissue removed from arthritic and control healthy joints (knees and ankles). Five sections were processed from each animal. As shown in Fig. 2, PNAd was strongly induced in a subset of vessels in arthritic synovium. These vessels co-stained with an antibody to CD31, a marker for vascular endothelium [36]. Normal tissue showed CD31^+ ^vessels, but there was no reactivity of any of the vessels with MECA-79. Frequent PNAd^+ ^vessels were observed in diseased synovium at all three time points, with staining intensity being slightly higher at day 3 as compared to day 15.

### GlcNAc6ST-2 is induced in PNAd^+ ^vessels in arthritic synovium

**Figure 3:**
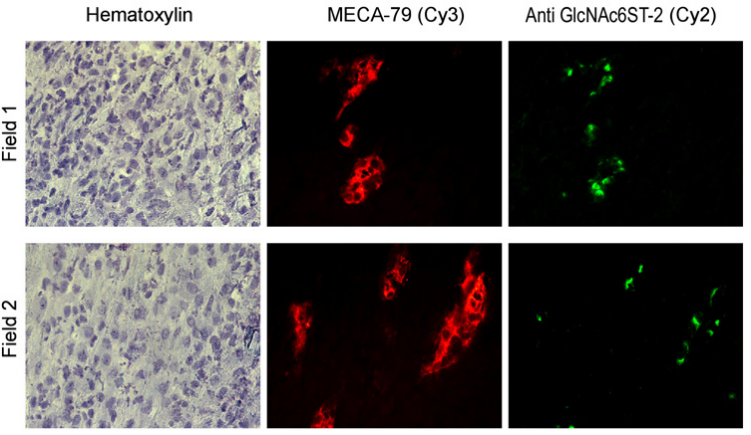
**Induction of GlcNAc6ST-2 in PNAd^+ ^vessels in arthritic synovium at day 3**. Sections of knee or ankle joint synovial tissue (obtained at days 3, 9, and 15 of the model) were stained with MECA-79 (Cy3, red), a GlcNAcST-2 antibody (Cy2, green) and with hematoxylin for bright field visualization. The depicted two-color fluorescence micrographs (400-fold magnification) were taken on sections from day 3 after disease onset and are representative for the other sections from this time. Staining patterns observed on sections from day 9 and day 15 were very similar to those from day 3.

Biosynthesis of the MECA-79 epitope (defining PNAd) in lymph node HEV depends on GlcNAc6ST-2 and to a lesser degree on GlcNAc6ST-1 [18,21,22]. The expression of PNAd in HEV-like vessels within ectopic lymphoid tissue and inflammatory lesions is associated with co-expression of GlcNAc6ST-2 and in at least one site, requires this enzyme [29,37]. Since we had detected PNAd^+ ^vessels in arthritic synovium (Fig. 2), we probed for GlcNAc6ST-2 protein in these vessels using a peptide-based antibody [29]. As shown in Fig. 3, GlcNAc6ST-2 was present in a punctuate Golgi-like pattern in PNAd^+ ^vessels, whereas GlcNAc6ST-2 protein was not detected in synovium from control animals (data not shown).

While in Figure 3 all of the vessels stained by MECA-79 also show at least one instance of punctuate Golgi-staining with the antibody to GlcNAc6ST-2, in other sections we observed a few vessels that exhibited punctuate GlcNAc6ST-2 staining in the absence of MECA-79 reactivity (data not shown). We also noted such a subset of GlcNAc6ST-2 positive but MECA-79 negative vessels in biopsies from human RA patients [25]. As MECA-79 reactivity depends not only on GlcNAc 6-O-sulfation but also on glycosyltransferases, such as the core 1 extension enzyme [38], such structures perhaps reflect a subset of "immature" vessels, which lack additional requirements for recognition by the MECA-79 antibody.

## Discussion

PNAd-expressing vessels, which often feature HEV morphology, have been reported to occur at sites of chronic inflammation (reviewed in references [6] and [28]), where they are believed to facilitate L-selectin-mediated recruitment of naïve and central memory lymphocytes and possibly other leukocytes to the lesions. Significantly, the presence of PNAd has been shown in synovial vessels in human RA [23-26] as well as in a model of Lyme disease arthritis in SCID mice [27]; however, its role in disease processes is unknown. Other than correlative histological evidence, there has been one report implicating L-selectin in the development of arthritis in an animal model [39]. However, a recombinant P-selectin antagonist, which can also bind to L-selectin, was shown to provide protection in the mouse CIA model [40].

Towards the objective of elucidating novel targets for RA therapies, the present study examined the expression of PNAd, and the sulfotransferases, which are involved in its elaboration, in a model of CIA. This model has proven to be predictive for therapeutic benefit in human disease [32].

Using the sulfation-dependent specific antibody MECA-79 in IF, we found PNAd expressed on a large number of synovial vessels in arthritic joints at three evenly spaced time points during the 15 day model but never on synovial vessels from healthy control joints. Furthermore, most of the PNAd^+^-positive vessels demonstrated a punctuate staining pattern for GlcNAc6ST-2 protein, consistent with a Golgi-localization.

In addition, we showed by quantitative PCR that transcripts encoding GlcNAc6ST-2, as well as for the related sulfotransferase GlcNAc6ST-1, were significantly upregulated in rheumatoid synovium as compared to healthy control tissue at all time points examined.

Our data demonstrate for the first time that de novo expression of PNAd together with induction of the endothelial sulfotransferases GlcNAc6ST-1 and -2 are associated with disease in the collagen-induced arthritis model, an established animal model of rheumatoid arthritis widely used in preclinical development of disease-modifying anti-rheumatic drugs (DMARDs). While potential roles of PNAd, as well as GlcNAc6ST-2, in RA have been considered before [25], it remains unclear, whether de novo expression of MECA-79-reactive ligands is causal rather than corollary to the disease process in RA. Significantly, Salmi and colleagues observed MECA-79^+ ^vessels in human arthritic joints; yet, because of the antibody's inability to significantly inhibit binding of activated mucosal immunoblasts to these vessels, the authors concluded that MECA-79-reactive ligands contributed little to disease-associated leukocyte recruitment [26]. In arthritic joints of SCID mice infected with *B. burgdorferi*, Schaible and coworkers observed the appearance of PNAd only late during infection following early upregulation of other adhesion molecules such as P-selectin, VCAM-1 and ICAM-1 [27]. Therefore these authors hypothesize that migration of leukocytes to inflamed joints in this model depends primarily on P-selectin-mediated rolling with subsequent engagement of α4β1/VCAM-1 and LFA-1/ICAM-1. However, we have recently demonstrated the pathophysiological significance of PNAd^+ ^vessels in a sheep model of bronchial asthma. Intravenous injection of MECA-79 prior to allergen challenge substantially blocked increases in airway resistance and airway hyperresponsiveness in allergic animals [41]. Use of the MECA-79 antibody in mouse in vivo models would be hampered by the very poor half-life of this rat IgM in the mouse circulation (S. Hemmerich, unpublished observations). Nevertheless, the observed induction of PNAd in the mouse CIA model should motivate alternative approaches to investigate the pathophysiological role of PNAd in RA. Recently, mice have become available which have the GlcNAc6ST-1 gene, the GlcNAc6ST-2 gene, or both inactivated [18,21,22]. The double-null mouse exhibits a complete loss of PNAd from lymph node HEV and a marked but not complete reduction of L-selectin ligand activity. Breeding of this mouse line onto a CIA-susceptible background such as B10RIII or DBA/J [33], should permit investigation of the contribution of PNAd to disease. Alternatively, the availability of an IgG-based antibody with a specificity related to MECA-79 but having improved pharmacokinetics would allow an antibody intervention approach to CIA in the mouse or other species. We and others have recently embarked on discovery of GlcNAc6ST and other sulfotransferase inhibitors by high throughput screening and rational drug design (reviewed in references [30], [42] and [43]). As the CIA model has become the most widely used preclinical animal model for DMARD development [31], positive proof of concept achieved in the target-validation studies discussed above might motivate the development of GlcNAc6ST inhibitors for disease-modifying treatment of RA in humans.

## Conclusion

MECA-79 binding glycoproteins defined as PNAd serve as ligands for L- selectin during the process of lymphocyte homing to lymph nodes. This class of ligands is also critical for inflammatory leukocyte trafficking and the associated disease, as recently demonstrated in a sheep model of asthma. Previous studies in mice have shown the requirement for the sulfotransferases GlcNAc6ST-1 and -2 in generating the MECA-79 epitope and the associated L-selectin ligand activity in HEV and HEV-like vessels. Immunohistochemical analyses have demonstrated ectopic expression of PNAd and GlcNAc6ST-2 in human rheumatoid arthritis. Further investigation into the functional role of PNAd and the requisite sulfotransferases in RA require a suitable animal model. Here we show, that PNAd is expressed de novo in synovial vessels in arthritic joints but not healthy control joints in the CIA model in mice. We further demonstrate that GlcNAc6ST-1 and GlcNAc6ST-2 transcripts are strongly upregulated in diseased tissue in this model and GlcNAc6ST-2 protein is present in a Golgi-associated pattern in the same vessels that show ectopic PNAd expression. Our data suggest the CIA model in mice for future studies into the functional role of PNAd-mediated leukocyte recruitment in RA and motivate the evaluation of PNAd antagonists or GlcNAc6ST inhibitors for therapeutic benefit in this widely used model of human RA.

## Methods

### Antibodies

The preparation of MECA-79 antibody and the peptide antibody against mouse GlcNAc6ST-2 are described elsewhere [29,41]. Control rat monoclonal IgM JG10.3 was a gift from Dr. Eugene Butcher (Stanford University). Rat anti mouse CD31 IgG2a (MEC13.3) and control rat IgG2a were from BD-Biosciences Pharmingen (San Diego, CA). Control rabbit IgG was from Sigma.

### Arthritis study

#### Animals

18 Male B10RIII mice (Jackson Labs, Bar Harbor, ME), 8 ± 1 weeks on arrival, at least 8 weeks at time of first immunization.

#### Study design and treatment groups

The in vivo study was carried out in its entirety at Bolder BioPATH, Boulder, CO. The study protocol was approved by Bolder BioPATH's Animal Care and Use Committee. Animals (2/group for arthritis, housed 1–2/cage, were acclimated for enough days after arrival to ensure that all mice were at least 8 weeks old. Disease group animals were anesthetized with isoflurane and injected intradermally with 200 μg bovine type II collagen (Elastin Products, Owensville, MO) emulsified in 100 μl Freund's complete adjuvant (Sigma-Aldrich, St. Louis, MO) with supplemental desiccated *Mycobacterium tuberculosis *H37 Ra (4 mg/ml, Difco Laboratories, Detroit, MI), while normal control group animals were un-manipulated. On days 21–26, onset of arthritis occurred and mice were randomized into groups (Table 1), once swelling was obviously established in at least one paw/joint. Animals were sacrificed and processed for necropsy as specified in Table 1.

**Table 1:** Disease and control groups in CIA study

Group	N	Description
1	1	Normal Control for TaqMan Q-PCR(Term and Necropsy Day 3 of Arthritis)
2	2	Disease Control for TaqMan Q-PCR (Term and Necropsy Day 3 of Arthritis)
3	1	Normal Control for IF sections (Term and Necropsy Day 3 of Arthritis)
4	2	Disease Control for IF sections (Term and Necropsy Day 3 of Arthritis)
5	1	Normal Control for TaqMan Q-PCR (Term and Necropsy Day 9 of Arthritis)
6	2	Disease Control for TaqMan Q-PCR (Term and Necropsy Day 9 of Arthritis)
7	1	Normal Control for IF sections (Term and Necropsy Day 9 of Arthritis)
8	2	Disease Control for IF sections (Term and Necropsy Day 9 of Arthritis)
9	1	Normal Control for TaqMan Q-PCR (Term and Necropsy Day 15 of Arthritis)
10	2	Disease Control for TaqMan Q-PCR (Term and Necropsy Day 15 of Arthritis)
11	1	Normal Control for IF sections (Term and Necropsy Day 15 of Arthritis)
12	2	DiseaseControl for IF sections (Term and Necropsy Day 15 of Arthritis)

#### Necropsy/processing of joints

At necropsy, samples of joint tissue were taken from Groups 1 through 12. Synovium was removed from the outside of the affected joints with periarticular skin (primarily knee and ankle). Synovium from around the patella of affected knees was also taken when possible. Additionally, individual digits showing clinical signs of arthritis were taken whole. All knee and ankle joints that had synovium samples removed were stored in 10% neutral buffered formalin for use in histology. Synovium specimens from Groups 1, 2, 5, 6, 9, and 10, which were chosen for TaqMan Q-PCR expression profiling, were snap frozen and shipped to Thios Pharmaceuticals. Synovium specimens from Groups 3, 4, 7, 8, 11, and 12 were snap-frozen in a "Gentle Jane" snap freezer (Alphelys, Plaisir, France) and stored at -70°C for Cryostat sectioning (10 slides per animal). Frozen sections were shipped on dry ice to Thios Pharmaceuticals for IF.

### Gene expression profiling by TaqMan real time polymerase chain reaction (Q-PCR)

The Taqman Q-PCR primers and probes (Table 2) for mouse GlcNAc6ST-1 and -2 were designed with PrimerQuest [44] and custom-ordered from Integrated DNA Technologies (Coralville, IA). The PCR efficiency and specificity of the primers was tested at the Genome Analysis Core Facility at the UCSF Comprehensive Cancer Center (San Francisco, CA) prior to use in the experiments.

**Table 2:** Primers and probes used for TaqMan Q-PCR

**mouse GlcNAc6ST-1 (GeneBank accession no. **NM_018763**):**	
forward primer	5' CAAGCGGCAGTTGGTGTATGTGTT 3',
reverse primer	5' TTCAGGGCAAACTGCTCCATTTCG 3',
Taqman probe:	5'ACAGTATGGCCAAGACGCTGCAAACA 3'.
	
**mouse GlcNAc6ST-2 (GeneBank accession no. **NM_011998**):**	
forward primer	5' ATCTTCTGCGTTCCGTCTTCCTGT 3',
reverse primer	5' TTCCTGGCGTTAGTATGGAAGGCA 3',
Taqman probe:	5'TGTGCTAGGGCAGCATTTGGAAACGA 3'.
	
**mouse GAPDH (GeneBank accession no. **NM_001001303**):**	
forward primer	5' GAATGGGAAGCTTGTCATCAACGG 3',
reverse primer	5' TAGACTCCACGACATACTCAGCAC 3',
Taqman probe:	5'AAGCCCATCACCATCTTCCAGGAGCGAGA 3'.
	
**mouse IL-1β (GeneBank accession no. **NM_008361**):**	
forward primer	5' GTACAAGGAGAACCAAGCAACGAC 3',
reverse primer	5' GTGCCGTCTTTCATTACACAGGAC 3',
Taqman probe:	5'ACCTGTGGCCTTGGGCCTCAAAGGAAAGAA 3'.
	
**mouse IL-10 (GeneBank accession no. **NM_010548**):**	
forward primer	5' ACTGCTATGCTGCCTGCTCTTACT 3',
reverse primer	5' TGGCCTTGTAGACACCTTGGTCTT 3',
Taqman probe:	5'AAGCATGGCCCAGAAATCAAGGAGCA 3'.

Prior to processing, synovia from both inflamed and control tissues designated for Q-PCR were brought to -20°C in RNA *later*-ICE (Ambion, Austin, TX) overnight, and homogenized with Tissuemiser (Fisher Scientific, Pittsburgh, PA) in Trizol (Invitrogen, Carlsbad, CA). Total RNA was isolated with Trizol following the manufacturer's instruction. Each cDNA was reverse transcribed from 0.5 μg total RNA (random hexamer priming) using the SuperScript III Platinum Two-Step qRT-PCR kit (Invitrogen) according to the manufacturer's instructions. Quantitative RT-PCR (40 cycles, 15 sec at 95°C, 30 sec at 52°C, 30 sec at 72°C, 1 sec at 82°C, read) was performed in triplicate at the Genome Analysis Core Facility at the UCSF Comprehensive Cancer Center using an ABI PRISM 7700 Sequence Detection System (ABI Biosystems, Foster City, CA). All data were normalized to the level of GAPDH transcripts in the same sample. The differences were considered significant when p < 0.05 (Student's T-test).

### Immunofluorescence on synovial tissue sections

Frozen tissue sections received on dry ice from Bolder BioPATH were thawed and brought to room temperature (5 sections per animal). The slides were fixed in ice-cold (0°C) acetone for 5 minutes, and then air-dried at ambient temperature for 30 minutes. Prior to staining, each tissue section was blocked with 5% mouse serum plus 3% bovine serum albumin in phosphate-buffered saline (blocking buffer). All antibodies were diluted in blocking buffer. The working concentration of primary antibodies (MECA-79, anti GlcNAc6ST-2 and anti CD31 and the corresponding isotype-matched control antibodies) was 1 μg/ml in all cases. Cy2 or Cy3 labeled secondary antibodies and streptavidin (Jackson ImmunoResearch Laboratories, West Grove, PA) were diluted according to the manufacturer's recommendations. All slides were double-stained either with MECA-79 and CD31 antibodies or MECA-79 and GlcNAc6ST-2 antibodies. PNAd expression was visualized with Cy3-labeled (red) goat anti-ratIgM while CD31 expression was visualized with Cy2-labeled (green) goat anti-ratIgG. GlcNAc6ST-2 expression was visualized with biotinylated goat anti-rabbit IgG followed by Cy2-labeled streptavidin. Two washings with phosphate buffered saline (PBS) were performed after each antibody incubation. Thereafter, slides were lightly counterstained for 2 seconds with Harris's hematoxylin and mounted with Fluosave (Calbiochem, San Diego, CA). Slides were examined under a Zeiss AxioVert200 microscope and images captured using AxioVision4 software (Carl Zeiss MicroImaging, Inc., Thornwood, NY).

## Abbreviations

*B. burgdorferi, Borrelia burgdorferi*; CIA, collagen-induced arthritis; DMARD, disease-modifying anti-rheumatic drug; GlcNAc6ST, N-acetylglucosamine 6-O-sulfotransferase; HEV, high endothelial venules; IBD, inflammatory bowel disease; IF, immunofluorescence; PNAd, peripheral node vascular addressin; Q-PCR, quantitative polymerase chain reaction; RA, rheumatoid arthritis; SCID mice, severe combined immunodeficient mice.

## Authors' contributions

JY designed and carried out the IF and Q-PCR studies. SDR participated and advised in design and coordination of the study, initiated and supervised the IF with GlcNAc6ST-2 antibodies, and helped in drafting the manuscript. PB had the in vivo portion of the study designed and executed at his institution. SH conceived of and supervised the study, participated in its design and coordination and drafted the manuscript. All authors read and approved the final manuscript.
